# *Perilla frutescens* as potential antimicrobial modifier to against forage oat silage spoilage

**DOI:** 10.3389/fmicb.2022.1053933

**Published:** 2022-12-20

**Authors:** Xiaomei Li, Fei Chen, Yi Xiong, Linna Guo, Jingjing Xu, Yanli Lin, Kuikui Ni, Fuyu Yang

**Affiliations:** ^1^College of Grassland Science and Technology, China Agricultural University, Beijing, China; ^2^College of Biological Engineering, Henan University of Technology, Zhengzhou, China; ^3^Beijing Sure Academy of Biosciences, Beijing, China

**Keywords:** *Perilla frutescens*, bacterial community, fungal community, fermentation quality, aerobic stability, forage oat

## Abstract

The aim of this study was to investigate the influence of *Perilla frutescens*, alone or in combination with *Lactobacillus plantarum* a214 or citric acid, on forage oat silage quality, bacterial and fungal microbiological profile during ensiling and aerobic exposure. With the exception of *Perilla frutescens*, all additives could improve silage quality of forage oat based on lower ammonia-nitrogen content and higher residual of water soluble carbohydrates during anaerobic fermentation compared to control silage, especially in *Perilla frutescens* combined with citric acid (CAPF). *Lactobacillus* was the dominant bacteria in all silages, while CAPF group increased the relative abundance of *Lactobacillus lindneri* and *Lactobacillus brevis* compared to control silage. The application of *Perilla frutescens* suppressed the relative abundance of yeasts such as *Pichia fermentans* and *Wickerhamomyces anomalus* in response to aerobic exposure, especially in CAPF treatment, leading to high acetic acids and lower dry matter loss, as well as good aerobic stability. Therefore, *Perilla frutescens*, alone or in combination with citric acid, has potential to improve aerobic stability of forage oat silage by shifting bacterial and fungal community composition, and can be used as new additive to prepare high-quality silage for animal production.

## Introduction

Forage oat (*Avena sativa* L.) is an important ruminant feed worldwide because of its good nutritive value and highly digestible fiber (Gomes et al., 2019; Jia et al., 2021), and ensiling has been considered to be one of the most efficient ways to conserve forage oat. However, a high level of water-soluble carbohydrate might result in higher fermentation losses and susceptible to aerobic spoilage (Cantoia Júnior et al., 2020). In particular, when silage is exposed to air, some species of yeast that survive in the ensiling process cause large nutrient losses (Zhao et al., 2018), and often compromise the feeding value and hygienic quality of the silage (Pahlow et al., 2003). Therefore, it is critical to search for an effective way to improve silage quality of forage oat.

The silage quality of forage oat mainly depends on the microorganisms and their metabolites. In response to aerobic exposure, microbial inoculum and chemical additives have been developed to modulate the microbial community to improve the silage quality and safety (Muck et al., 2018). *Lactobacillus plantarum* was shown to promote lactic fermentation and reduce pH, thereby improving silage anaerobic fermentation (Muck et al., 2018). In addition, citric acid, a weak organic acid, could enhance fermentation quality by lowering pH and proteolysis (Tao et al., 2020). However, both of them were failed to improve aerobic stability of ensiling (Muck et al., 2018; Tao et al., 2020). In recent years, phenolic and flavonoid compounds have been reported to have inhibitory activity against undesirable microorganisms such as *Enterobacter*, *Clostridium*, fungi such as *Candida*, *Aspergillus*, and so on (Zacchino et al., 2017; Chan et al., 2018). Furthermore, medicinal plants or its secondary metabolites have been used as ideal, safe and novel additives for improving food security and animal production due to their highly inhibitory effects on bacteria and fungi (Kang et al., 2019; Falleh et al., 2020; Li et al., 2021b). However, little is known about the use of herbs and plants high in bioactive substances in the ensiling of forage oat.

Purple perilla (*Perilla frutescens* L.), an annual short-day medicine food homology plant that belongs to the family *Lamiaceae* and the genus *Perilla*, is commonly available in many countries and has been cultivated in China for more than 2,000 years. With a large variety of functional ingredients such as essential oil, polyphenol, flavone and so on, purple perilla shows antimicrobial, antioxidants and healthy functions (Lv et al., 2011). The stems and leaves of purple perilla contain 0.3%–0.7% essential oil, and the primary components are terpenoids including perilla ketone, perilla aldehyde, perilla alcohol, α-pinene, β-pinene (Lim and Shin, 2014; Ahmed and Al-Zubaidy, 2020), and exert potent antifungal properties. In particular, the stable low-acidic environment enhanced the antimicrobial activity of terpenes, phenols and so on (Reddy et al., 2020). Both *Lactobacillus plantarum* and citric acid, as preferred silage additives, could accelerate the acidification of the silage environment indirectly or directly (Muck et al., 2018; Mugabe et al., 2020). Furthermore, *Lactobacillus*-mediated fermentation could lead to the liberation or synthesis of bioactive compounds in herbal plants, and enhance their antimicrobial activity (Hussain et al., 2016). On the other hand, organic acids and essential oils work synergistically and produce greater effects. The essential oils could disrupt cell membrane integrity, thereby making microbes more vulnerable to the acidic environment caused by organic acids (Karatzas et al., 2001; Reddy et al., 2020). However, how purple perilla, especially in combination with *L. plantarum* or citric acid, influences the bacterial and fungal community of silage remains unclear.

Thus, the objective of this study was to evaluate the effects of *P. frutescens* and its combination with *L. plantarum* or citric acid on forage oat silage fermentation, bacterial and fungal microbiological profile during ensiling, and aerobic exposure. We hypothesized that *P. frutescens* combined with *L. plantarum* or citric acid would have a positive synergistic effect on improving silage fermentation as well as aerobic stability.

## Materials and methods

### Silage preparation

Whole forage oat (*Avena sativa* L., Magnum) was manually collected from the farm of China Agricultural University (Zhangye, Gansu, China) on 23 August 2020. The lactic acid bacteria inoculant, purple perilla, and citric acid were selected as additives in this study. The inoculant *L. plantarum* a214 used in this study could grow at low pH and produce higher-quality local grass silage (Li et al., 2018). The dried whole purple perilla was obtained from Beijing Tongrentang Co., Ltd., China, and milled through a 60-mesh screen by a multimill. Citric acid (CAS: 77-92-9, purity > 99%) was obtained from Aladdin Industrial Inc., Shanghai, China.

The fresh oat forage was harvested at approximately 5 cm above the ground, and then directly chopped into 2 cm pieces using a forage crusher (600 mm, Shandong Anke Hardware Tools Co., Ltd., Shandong, China). Six different treatments were conducted as follows: (1) control (no additive, CK), (2) *L. plantarum* a214 (LP), (3) 2% citric acid (CA), (4) 2.5% purple perilla (PF), (5) the combination of 2.5% purple perilla and *L. plantarum* a214 (LPPF), and (6) the combination of 2.5% purple perilla and 2% citric acid (CAPF). According to the manufacturers guidelines, *L. plantarum* a214 and citric acid were dissolved in sterile distilled water to an equivalent of 10^6^ colony-forming unit (cfu)/g and 20 g/kg of fresh matter (FM), respectively. The application rate of purple perilla was 25 g/kg of FM. After mixing thoroughly, approximately 4 kg of the oat forage from each treatment was packed into a polyethylene plastic bag (45 cm × 75 cm, Suqian Xizhao Trading Co., Ltd., Jiangsu, China) in triplicate. The bags were vacuum-sealed *via* vacuum sealing machine (DZ-600/2S, Wenzhou Kaichi Packing Machinery Co. Ltd., Zhejiang, China) and then stored at room temperature (about 22°C–26°C). After 60 days of ensiling, the silage bag from each treatment was opened, and samples were collected immediately for measurement of microorganisms, fermentation parameters, and chemical composition. Two 20-g subsamples from each homogenized sample were quickly placed into 50 ml sterile centrifuge tubes and stored into −80°C freezers for analysis of bacteria and fungi community.

### Aerobic stability

To evaluate aerobic stability, the whole forage oat silages ensiled for 60 days were quickly placed in sterile plastic buckets (2 l capacity, Hewanglan paper and plastic products factory, Beijing, China) with a density of approximately 350 kg/m^3^. All plastic buckets were covered with 2 layers of sterile cheesecloth and stored at an ambient temperature (22°C–26°C). A thermocouple wire was placed in the geometric center of each plastic buckets, and real-time temperatures were measured every 5 min using a data logger (MDL-1048A; Shanghai tianhe automation instrument Co., Ltd., Shanghai, China). After 3 days of aerobic exposure, silage of each plastic bucket was weighted, thoroughly mixed, and sampled to analyze fermentation quality and microbes. The aerobic stability was defined based on the time when the temperature of silage had increased by 2°C above the ambient temperature.

### Fermentation, chemical composition, and microbial analyses

Twenty grams of silage sample was homogenized in a blender for 1 min with 180 ml of sterile saline water, and filtered by filter paper. The pH value of extract was measured immediately using a portable pH meter (PHS-3C, INESA Scientific Instrument, Shanghai, China). A portion of filtrate was centrifuged at 10,000 ×g for 10 min at 4°C (5810R; Eppendoff, Hamburg, Germany), and then filtrated through 0.22 μm membrane filters for organic acid analysis as described by Yan et al. (2019). The ammonia-nitrogen (NH_3_-N) content was determined through phenol-hypochlorite method. According to Li et al. (2021a), another 20 g samples were mixed with 180 ml sterilized saline and shaken in a thermostatic oscillation incubator at 160 r/min, 4°C for 30 min. Fungi (molds and yeasts) were cultivated aerobically using Rose Bengal agar at 28°C for 48 h. Lactic acid bacteria counts were measured *via* Man Rogosa Sharpe agar at 30°C for 48 h anaerobically. Coliform bacteria were cultivated *via* blue light agar (Nissui) for 48 h at 37°C.

Both fresh and silage samples were dried at 65°C for 3 days in a forced-air oven to determine dry matter (DM) content. Then, all samples were ground and passed through a 1-mm sieve for further chemical analysis. Crude protein (CP) content was determined by the Kjeldahl method (Hasan, 2015). Acid detergent fiber (ADF) and neutral detergent fiber (NDF) contents were analyzed using the ANKOM 200 system according to the manufacturer’s instructions. Water-soluble carbohydrate (WSC) was estimated according to the methodology of Thomas (1977). Dry matter loss was calculated according to weight differences between pre-and post-ensiling.

### Microbial analysis

Total bacterial or fungal genomic DNA from silage samples was extracted *via* a DNA Kit (DP302-02, Tiangen, Beijing, China) according to the manufacturer’s guidance. The purity and concentration of the extracted DNA were determined as specifically described by Yan et al. (2019). The PCR amplification of the full-length 16S rRNA gene were using the specific primers 27F (5′-GAGAGTTTGATCCTGGCTCAG-3′) and the reverse primer 1492R (5′-TACCTTGTTACGACTT-3′). The PCR procedures were followed as previously described by (Li et al., 2021a). Fungal full-length ITS genes were amplified using the primers ITS1F (5′-CTTGGTCATTTAGAGGAAGTAA-3′) and the reverse primer ITS4 (5′-TCCTCCGCTTATTGATATGC-3′). The PCR program for ITS was as follows: 95°C for 5 min; 8 cycles of 95°C for 1 min, 55°C for 30 s, and 72°C for 45 s; 24 cycles of 95°C for 1 min, 60°C for 30 s, and 72°C for 45 s, with a final extension of 72°C for 7 min. The 16S rRNA and ITS libraries and amplicon sequencing were carried out with PacBio Template Prep Kit (Pacific Biosciences of California, Inc., California, America) and Sequel instrument (ABI GeneAmp® 9700), respectively.

Raw Circular Consensus Sequencing reads were demultiplexed, quality filtered, optimization, and analyzed using QIIME 1.9.1 and the Greengenes 13.8, UNITE fungal ITS reference database according to the report of Romero et al. (2018) and Drouin et al. (2021). Subsequently, bacterial and fungal OTUs were assigned using the Ribosomal Database Project classifier *via* the Amplicon Tagger 16S and ITS training sets, respectively (Drouin et al., 2021). Alpha diversity indices, including Chao1 and Shannon index were calculated using QIIME software. The principal component analysis (PCA) was calculated in QIIME to assess the structural variation of microbiota. LEfSe analysis were conducted using a free online platform.^1^

### Statistical analysis

Analysis of variance (ANOVA) was conducted using general linear model in SPSS (version 19.0) to determine the significant difference among samples. The level of statistical significance was set to *p* < 0.05. The data from chemical composition, fermentation characteristics, microbial population, and alpha diversity of the silages were subjected to two-way ANOVA with a fully randomized design, with aerobic exposure days (days) and additives (Add) as the main variables. Mean values were compared using Tukey’s test.

## Results

### Chemical composition of raw materials

The chemical composition of raw materials is shown in Table 1. The DM content of forage oat was 302.10 g/kg of fresh matter, and its CP, NDF, WSC contents were 86.48, 582.60 and 115.25 g/kg on a DM basis, respectively. The DM, CP, NDF and WSC contents of dried purple perilla were 932.00, 226.55, 300.97, and 138.29 g/kg DM, respectively.

**Table 1:** Chemical composition of raw materials.

Item	Whole forage oat	Dried purple perilla
DM (g/kg)	302.10 ± 2.35	932.00 ± 3.56
Crude protein (g/kg DM)	86.48 ± 1.73	226.55 ± 0.70
Neutral detergent fiber (g/kg DM)	582.60 ± 6.85	300.97 ± 4.91
Acid detergent fiber (g/kg DM)	322.96 ± 4.24	213.85 ± 3.88
Hemicellulose (g/kg DM)	259.64 ± 4.02	87.12 ± 2.71
Water-soluble carbohydrate (g/kg DM)	115.25 ± 2.30	138.29 ± 0.76

Data are means of three samples. DM, dry matter.

### Fermentation characteristics and microbial numbers of silages

The fermentation end products and microbial numbers of oat silage after 60 days of ensiling and after exposure to air are shown in Table 2. There was an interaction (*p* < 0.01) between the effects of fermentation day and additive for pH because it was higher after 3 days of aerobic exposure than 60 days of ensiling for LP, CA, and LPPF treatments, but it was similar for CK, PF, and CAPF in both days. We also found an interaction (*p* < 0.01) between the application of additive and fermentation day for lactic acid because the contents of lactic acid in CK, CA, LP, and LPPF groups sharply decreased after exposure to air compared with 60 days of ensiling. In addition, treatments with CA and CAPF resulted in lower concentration of lactic acid compared with CK after 60 days of fermentation. The application of PF and CAPF caused a higher (*p* < 0.01) acetic acid concentration when compared with other treatment, and 60 days of ensiling was higher (*p* < 0.01) in acetic acid than exposure to air. The concentration of propionic acid was not affected by fermentation days (*p* = 0.742), but the application of purple peril (PF, LPPF, and CAPF) had a higher (*p* < 0.01) concentration of propionic acid than other treatments.

**Table 2:** Fermentation characteristics and microbial counts of forage oat silages during aerobic exposure.

Item	pH	Lactic acid (g/kg DM)	Acetic acid (g/kg DM)	Propionic acid (g/kg DM)	Lactic acid bacteria (log_10_ cfu/g FM)	Molds (log_10_ cfu/g FM)	Yeasts (log_10_ cfu/g FM)
**60 days of fermentation**							
CK	4.02^bcd^	69.10^d^	10.54	1.67	5.69^b^	<2.00^a^	<2.00^a^
LP	3.86^ab^	82.37^d^	4.48	3.25	5.17^a^	<2.00^a^	2.34^b^
CA	3.67^a^	48.03^bc^	13.16	3.51	5.26^a^	<2.00^a^	3.31^c^
PF	4.13^bcd^	66.49^cd^	13.05	12.47	5.97^bc^	<2.00^a^	<2.00^a^
LPPF	3.89^abc^	66.77^cd^	6.99	9.26	5.06^a^	<2.00^a^	2.13^b^
CAPF	3.93^bc^	32.56^b^	15.45	11.79	6.17^cd^	<2.00^a^	<2.00^a^
**3 days of aerobic exposure**							
CK	4.06^bcd^	32.00^b^	1.59	2.29	5.95^bc^	4.66^b^	3.60^c^
LP	4.32^ef^	43.40^b^	1.40	2.96	7.36^e^	6.72^c^	6.82^d^
CA	4.25^de^	8.49^a^	0.90	3.95	7.22^e^	< 2.00^a^	7.33^d^
PF	4.12^bcd^	64.86^cd^	10.95	9.15	6.45^d^	<2.00^a^	<2.00^a^
LPPF	4.52^f^	47.02^bc^	0.97	9.77	7.44^e^	7.18^d^	7.46^d^
CAPF	3.95^bc^	28.90^b^	15.16	12.46	5.86^bc^	<2.00^a^	<2.00^a^
**Day means**							
60 days	3.92	60.89	10.61^b^	6.99	5.55	0.00	1.30
3 days	4.20	37.45	5.16^a^	6.76	6.71	3.09	4.20
**Additives means**							
CK	4.03	50.55	6.07^a^	1.98^a^	5.82	2.33	1.80
LP	4.09	62.89	2.94^a^	3.11^a^	6.27	3.36	4.58
CA	3.96	28.26	7.03^a^	3.73^a^	6.24	0.00	5.32
PF	4.13	65.68	12.00^b^	10.81^b^	6.21	0.00	0.00
LPPF	4.21	56.90	3.98^a^	9.52^b^	6.25	3.59	4.80
CAPF	3.94	30.73	15.31^b^	12.13^b^	6.02	0.00	0.00
**SEM**	0.03	4.42	1.2	1.08	0.11	0	0.33
**Effects and interactions (*p*-value)**							
Add	<0.001	<0.001	<0.001	<0.001	<0.001	<0.001	<0.001
Days	0.015	<0.001	<0.001	0.742	0.029	<0.001	<0.001
Add × days	0.001	0.009	0.102	0.54	<0.001	<0.001	<0.001

^a–f^Means of additives treatments within a column with different superscripts differ followed are statistically different (*p* < 0.05). CK, control; LP, Lactobacillus plantarum a214; CA, citric acid; PF, purple perilla; LPPF, purple perilla + Lactobacillus plantarum a214; CAPF, purple perilla + citric acid; SEM, standard error of means; DM, dry matter; H, additives; T, aerobic exposure days; d, days. H **×** T, the interaction between silage additives and aerobic exposure day.

**Figure 1:**
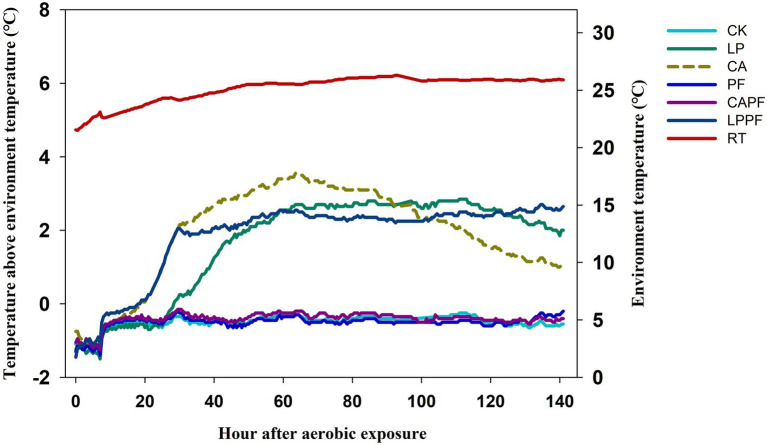
Temperature after aerobic exposure of forage oat silages treated additives. CK, control; LP, *Lactobacillus plantarum a*214; CA, citric acid; PF, purple perilla; LPPF, purple perilla + *Lactobacillus plantarum* a214; CAPF, purple perilla + citric acid; RT, room temperature.

The high levels of lactic acid bacteria (>5 log_10_ cfu/g FM) were observed in all silages during both ensiling and aerobic exposure. An interaction (*p* < 0.01) was found between fermentation day and additive for the numbers of molds and yeasts. Molds were not detected in all silage after 60 days of ensiling (<2 log_10_ cfu/g FM), but its number increased markedly (*p* < 0.01) in CK, LP, and LPPF when exposed to air. Number of yeast in CK, LP, CA, and LPPF (3.60~7.46 log_10_ cfu/g FM) were higher (*p* < 0.01) in 3 days of exposure compared to 60 days of ensiling, but no yeast was detected in PF and CAPF for both days (<2 log_10_ cfu/g FM). As presented in Figure 1, the PF, PF + CA, and CK treatments had a good aerobic stability, and the aerobic stability exceeded 144 h The application of CA, PF + LP, and LP accelerated the aerobic spoilage of oat silage and remained stable only for 29, 29.5, and 48.5 h, respectively.

Table 3 shows the DM loss and chemical composition of forage oat silage. There was an interaction (*p* < 0.01) between fermentation day and additive for DM loss because it was significantly higher in 3 days of aerobic exposure than 60 days of ensiling for CK, LP, CA, and LPPF treatments, but CAPF had the lowest (*p* < 0.01) DM loss (average 17.41 g/kg DM) than other treatment in both days. The application of purple peril (PF, LPPF and CAPF) caused a higher (*p* < 0.01) DM content than other treatments in both days. We also found an interaction (*p* < 0.01) between the application of additive and fermentation day for NH_3_-N/TN because all additives resulted in lower (*p* < 0.01) NH_3_-N/TN compared with CK, except for the PF treatment. The content of CP and NDF were unaffected by fermentation days, but PF and LPPF resulted in higher (*p* < 0.01) concentration of CP compared with other treatments. There were minor differences in the concentration of ADF among additives except with LP treatment. In addition, an interaction (*p* < 0.01) was found between fermentation day and additive for WSC. On 60 days of ensiling, LP and CAPF remained significantly highest levels of WSC residues (>70.00 g/kg DM) compared to others, but the difference among treatments were small in 3 days of aerobic exposure.

**Table 3:** Chemical compositions of forage oat silages during aerobic exposure.

Item	DM (g/kg)	DM loss (g/kg DM)	NH_3_-N/TN (%)	Crude protein (g/kg DM)	NDF (g/kg DM)	ADF (g/kg DM)	WSC (g/kg DM)
**60 days of fermentation**							
CK	284.35^cd^	59.87^cd^	12.34^d^	91.68	555.57	295.47	24.28^ab^
LP	292.47^e^	32.48^ab^	2.92^abc^	81.15	579.12	326.63	74.99^e^
CA	286.76^de^	51.75^bc^	5.35^bc^	87.90	555.34	310.04	53.02^d^
PF	313.96^gh^	60.83^cd^	12.81^d^	100.61	553.20	309.92	32.05^abc^
LPPF	317.86^h^	47.70^bc^	3.25^abc^	102.23	527.19	289.75	61.37^d^
CAPF	331.42^J^	13.37^a^	4.16^abc^	92.01	522.10	297.66	77.70^e^
**3 days of aerobic exposure**							
CK	278.07^bc^	81.04^e^	19.53^e^	91.30	537.55	284.79	22.41^a^
LP	274.97^ab^	91.50^e^	2.05^ab^	87.65	583.24	320.30	39.72^c^
CA	268.75^a^	112.49^f^	3.38^abc^	91.60	527.63	290.97	24.66^ab^
PF	310.91^g^	63.52^cd^	15.12^e^	103.49	512.80	291.71	34.07^bc^
LPPF	299.74^f^	97.16^ef^	1.27^a^	106.63	526.00	285.14	52.69^d^
CAPF	324.88^I^	21.44^a^	6.16^c^	96.75	502.13	258.48	31.35^abc^
**Day means**							
60 days	304.47	44.33	7.19	92.60	548.75	304.91^b^	53.90
3 days	292.89	77.86	7.53	96.24	531.56	288.57^a^	34.15
**Additives means**							
CK	281.21	70.46	15.94	91.49^b^	546.56^b^	290.13^ab^	23.35
LP	283.72	61.99	2.49	84.40^a^	581.18^c^	323.47^c^	57.36
CA	277.76	82.12	4.37	89.75^ab^	541.49^ab^	300.51^b^	38.84
PF	312.44	62.18	13.97	102.05^c^	533.00^ab^	300.82^b^	33.06
LPPF	308.80	72.43	2.26	104.43^c^	526.60^ab^	287.45^ab^	57.03
CAPF	328.15	17.41	5.16	94.38^b^	512.12^a^	278.07^a^	54.53
SEM							
**Effects and interactions (*P*-value)**							
Add	<0.001	<0.001	<0.001	0.001	0.002	0.001	<0.001
Days	<0.001	<0.001	<0.001	0.060	0.051	0.004	<0.001
Add × days	0.003	0.002	0.001	0.796	0.663	0.448	<0.001

^a–i^Means of additives treatments within a column with different superscripts differ followed are statistically different (*p* < 0.05). CK, control; LP, Lactobacillus plantarum a214; CA, citric acid; PF, purple perilla; LPPF, purple perilla + Lactobacillus plantarum a214; CAPF, purple perilla + citric acid; SEM, standard error of means; DM, dry matter; NDF, neutral detergent fiber; ADF, acid detergent fiber; WSC, water-soluble carbohydrate; NH_3_-N/TN, the ratio of ammoniacal nitrogen to total nitrogen; H, additives; T, aerobic exposure days; d, days. H **×** T, the interaction between silage additives and aerobic exposure day.

### Microbial silage diversity

**Figure 2:**
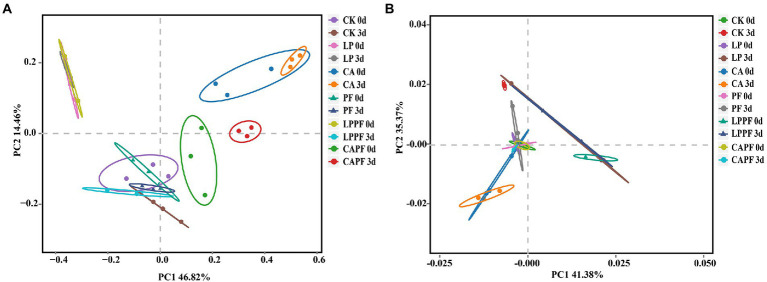
Principal component analysis of the bacterial **(A)**, and fungal community **(B)**, of forage oat silages. CK, control; LP, *Lactobacillus plantarum a*214; CA, citric acid; PF, purple perilla; LPPF, purple perilla + *Lactobacillus plantarum* a214; CAPF, purple perilla + citric acid; 0d, 60-day of ensiling; 3 d, 3 days of aerobic exposure.

The microbial diversity of forage oat silages is shown in Table 4. The coverage value of all samples were approximately 0.99. For bacteria, the application of CA showed higher (*p* < 0.01) value of OTUs and Chao 1 when compared to CK. Besides, there was a trend for Shannon index to be higher in 3 days of exposure than in 60 days of ensiling, especially in LPPF and CAPF. For fungi, there was an interaction (*p* < 0.01) between fermentation day and additive for OTUs and Shannon index because they were lower (*p* < 0.01) after 3 days of aerobic exposure than 60 days of ensiling for CK, LP and CA treatments. On 3 days of aerobic exposure, PF and CAPF resulted in higher (*p* < 0.01) value of OTUs and Shannon index compare with other treatment. Treatment with LPPF resulted in lower (*p* < 0.01) Chao 1 compared to CK, while treatment with CAPF resulted in higher (*p* < 0.01) Chao 1. As showed in PCA analysis (Figure 2), bacteria community of forage oat silages were more influenced by additives than ensiling time. The bacterial communities in all citric acid treated groups and *L. plantarum* a214 treated groups were clearly separated from other groups, except for LPPF. After 3 days of aerobic exposure, the fungal communities of the PF and CAPF groups, LP and LPPF groups, CA group, and CK group were clearly distinguished.

**Table 4:** The microbial diversity of forage oat silages during aerobic exposure.

Sample ID	Bacteria				Fungi			
	OUTs	Shannon	Chao1	Coverage	OUTs	Shannon	Chao1	Coverage
**60 days of fermentation**								
CK	33.67	2.18^cd^	46.07	0.99	123.00^e^	4.72^c^	131.20	0.99
LP	36.33	0.16^a^	45.95	0.99	123.00^e^	4.43^c^	134.56	0.99
CA	73.67	1.42^b^	90.75	0.99	75.00^cd^	2.27^b^	84.33	0.99
PF	44.67	2.29^cd^	62.23	0.99	108.33^de^	4.30^c^	117.69	0.99
LPPF	66.33	0.43^a^	86.25	0.99	51.00^abc^	0.72^a^	70.61	0.99
CAPF	47.67	1.82^bc^	56.38	0.99	148.33^e^	4.53^c^	166.24	0.99
**3 days of aerobic exposure**								
CK	54.67	2.38^cd^	66.73	0.99	61.00^bc^	0.83^a^	72.14	0.99
LP	45.00	0.18^a^	64.19	0.99	27.00^ab^	0.32^a^	47.73	0.99
CA	92.33	2.03^bc^	96.33	0.99	38.33^ab^	0.63^a^	71.78	0.99
PF	93.67	2.55^d^	112.55	0.99	117.00^de^	3.65^c^	135.92	0.99
LPPF	72.33	2.13^cd^	85.49	0.99	11.33^a^	0.83^a^	29.44	0.99
CAPF	83.67	2.57^d^	94.12	0.99	137.67^e^	4.64^c^	146.18	0.99
**Day means**								
60 days	84.50^a^	1.38	64.61	-	104.78	3.50	117.44^b^	-
3 days	135.5^b^	1.97	86.56	-	65.39	1.82	83.87^a^	-
**Additives means**								
CK	44.17^a^	2.28	56.40^a^	-	92.00	2.78	101.67^bc^	-
LP	40.67^a^	0.17	55.07^a^	-	75.00	2.38	91.15^b^	-
CA	83.00^b^	1.73	93.54^b^	-	56.67	1.45	78.06^ab^	-
PF	69.17^ab^	2.42	87.39^ab^	-	112.67	3.98	126.81^cd^	-
LPPF	69.33^ab^	1.28	85.87^ab^	-	21.17	0.78	50.03^a^	-
CAPF	65.67^ab^	2.19	75.25^ab^	-	143.00	4.59	156.21^d^	-
SEM				-				-
**Effects and interactions (*p*-value)**								
Add	0.005	<0.001	0.021	-	<0.001	<0.001	0.002	-
Days	0.026	<0.001	0.078	-	<0.001	<0.001	<0.001	-
Add × days	0.562	0.004	0.573	-	0.014	<0.001	0.055	-

^a–e^Means of additives treatments within a column with different superscripts differ followed are statistically different (*p* < 0.05).CK, control; LP, Lactobacillus plantarum a214; CA, citric acid; PF, purple perilla; LPPF, purple perilla + Lactobacillus plantarum a214; CAPF, purple perilla + citric acid.

### Dynamic changes of bacteria communities

**Figure 3:**
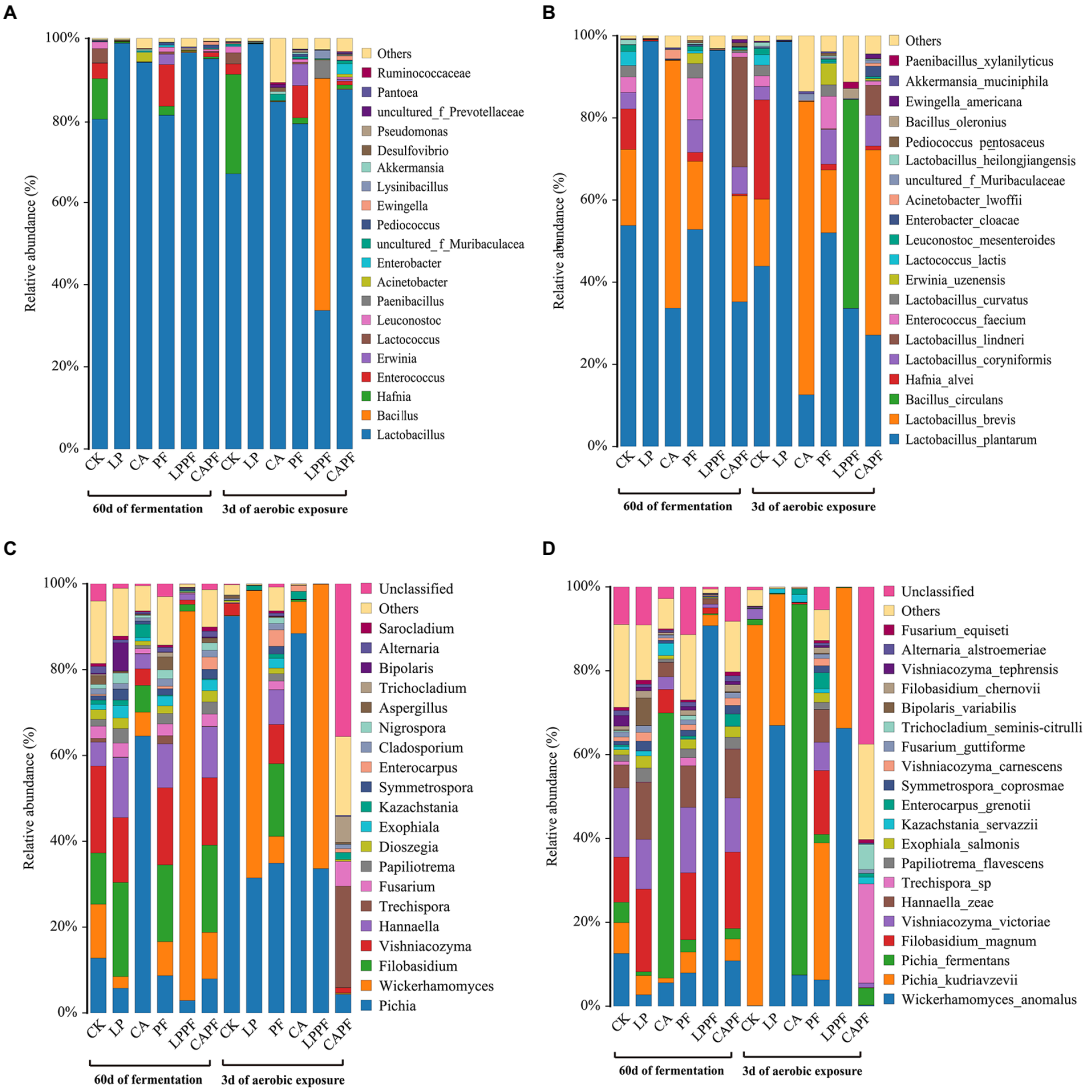
Relative abundances of the silage bacterial and fungal composition. Bacteria at genus level **(A)**, and species levels **(B)**; fungal at genus level **(C)**, and species levels **(D)**; CK, control; LP, *Lactobacillus plantarum* a214; CA, citric acid; PF, purple perilla; LPPF, purple perilla + *Lactobacillus plantarum* a214; CAPF, purple perilla + citric acid.

**Figure 4:**
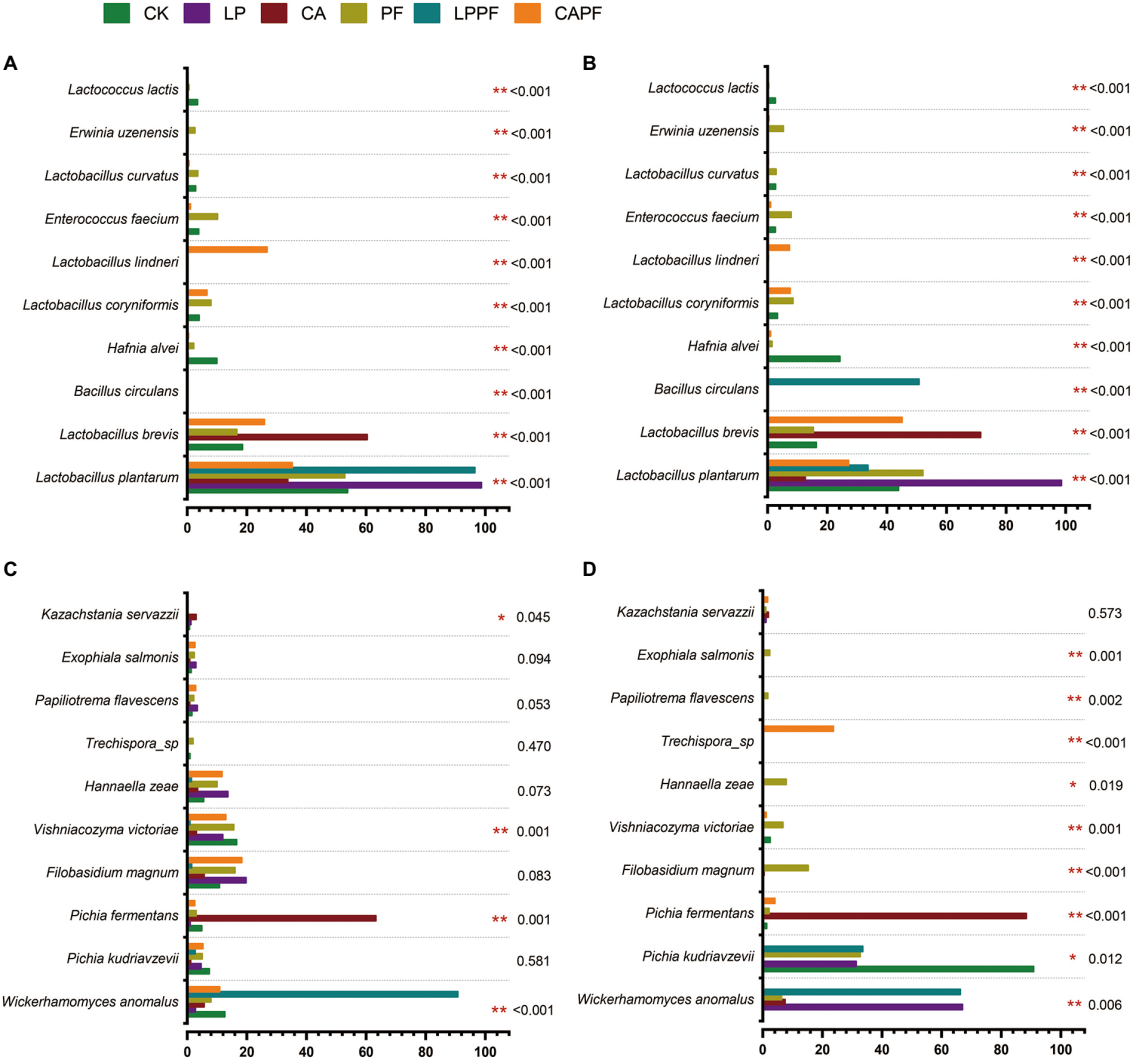
One-way analysis of variance bar plots of top 10 species of bacterial and fungal in forage oat silages. Bacteria species level on 60-day of ensiling **(A)**, and 3 days of aerobic exposure **(B)**; fungal species level on 60-day of ensiling **(C)**, and 3 days of aerobic exposure **(D)**. * and ** represent *p* < 0.05 and *p* < 0.01, respectively. CK, control; LP, *Lactobacillus plantarum* a214; CA, citric acid; PF, purple perilla; LPPF, purple perilla + *Lactobacillus plantarum* a214; CAPF, purple perilla + citric acid.

Relative abundances of forage oat silage bacterial composition during ensiling and aerobic exposure are shown in Figures 3, 4. Overall, *Lactobacillus* was the most abundant genus in all samples following 60 days of ensiling, with an abundance of more than 80% (Figures 3A, 4A). The application of *L. plantarum* a214 and citric acid significantly increased the abundance of *Lactobacillus* (94.12%~98.80%) compared with CK. Once exposed to aerobic exposure, *Lactobacillus* remained the predominant bacteria, while its abundance decreased dramatically in CK, CA, and LPPF groups compared with 60 days of ensiling. Instead, an increase in the proportion of *Hafnia* and *Bacillus* occurred in CK and LPPF groups, respectively.

**Figure 5:**
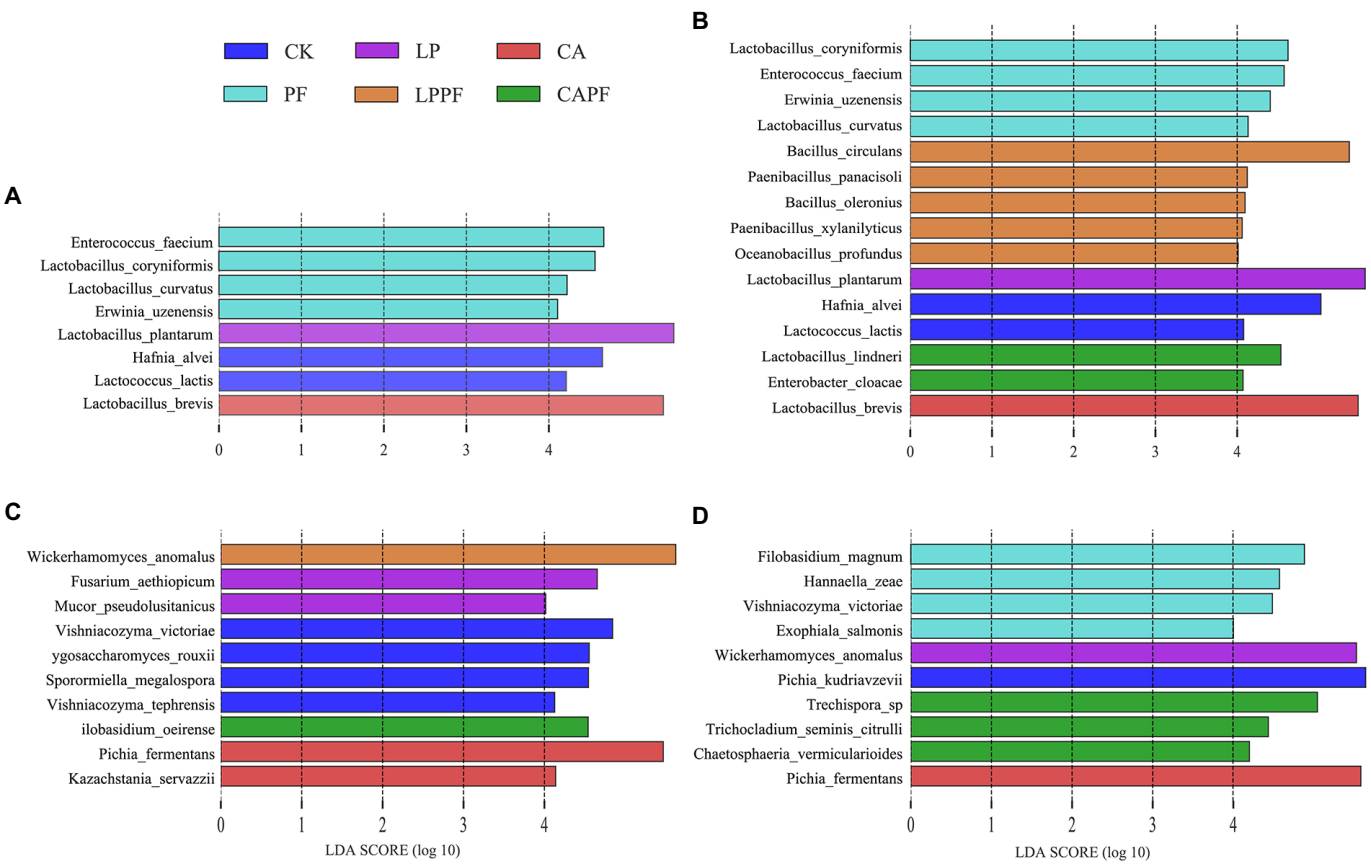
LEfSe analysis of bacterial and fungal variations in forage oat silages. Bacteria species level on 60-day of ensiling **(A)**, and 3 days of aerobic exposure **(B)**; fungal species level on 60-day of ensiling **(C)**, and 3 days of aerobic exposure **(D)**. CK, control; LP, *Lactobacillus plantarum* a214; CA, citric acid; PF, purple perilla; LPPF, purple perilla + *Lactobacillus plantarum* a214; CAPF, purple perilla + citric acid.

At species levels (Figures 3B, 4B), *L. plantarum* was the most abundant bacteria in CK, LP, PF, and LPPF groups after 60 days of ensiling. Especially, *L. plantarum* was also identified as the most representative bacterium in all *L. plantarum* a214 treated silages (Figure 5A), its abundance exceeded 96.42%. Whereas the predominant bacteria in CA groups were *Lactobacillus brevis* (60.33%) and *L. plantarum* (33.67%). It is noteworthy that three *Lactobacillus* species with similar relative abundance were found in CAPF groups, including *L. plantarum* (35.22%), *L. brevis* (25.82%), *Lactobacillus lindneri* (26.73%). Compared with 60 days of fermentation, there were no significant bacteria changes in LP and PF groups, while the abundance of *L. plantarum* dramatically decreased in CK, CA, LPPF and CAPF after 3 days of aerobic exposure. A high abundance of *Hafnia alvei* (24.19%) occurred in CK groups, and it could be used as a biomarker in CK by LEfSe algorithm (Figures 5A,B). Besides, CAPF groups showed a higher abundance of *L. brevis* (45.07%) and *Lactobacillus coryniformis* (7.51%) compared with CK.

### Dynamic changes of fungal communities

Relative abundances of forage oat silage fungal composition during ensiling and aerobic exposure are shown in Figures 3, 4. During 60 days of ensiling, *Pichia*, *Wickerhamomyces*, *Filobasidium*, *Vishniacozyma*, and *Hannaella* were the predominant fungal genus among CK, LP, PF, and CAPF groups (Figures 3C, 4C). *Pichia* was the most predominant fungi in CA group accounting for 64.52%, whereas *Wickerhamomyces* (90.74%) dominated the LPPF group. Upon exposure to air, the abundance of *Pichia* increased dramatically in all silages (except for CAPF groups), especially in CK and CA groups, accounting for 92.48% and 88.43%, respectively. *Wickerhamomyces* and *Pichia* were the main genus in all *L. plantarum* a214 treated silages at 3 days of exposure. Interestingly, *Trechispora* replaced other genus as the dominant population fungi in CAPF group compared to 60 days of ensiling.

At species levels (Figures 3D, 4D), during 60 days of ensiling, *Wickerhamomyces anomalus*, *Filobasidium magnum*, *Vishniacozyma victoriae*, *Pichia kudriavzevii* and *Hannaella zeae* were the main fungal species among CK, LP, PF, CAPF groups. The application of LPPF and CA increased the abundance of *W. anomalus* and *Pichia fermentan*s, respectively (Figure 5C). Once aerobic exposure, *P. kudriavzevii* was the most significantly discriminating in CK group (Figure 5D), its abundance increased from 7.37 to 90.81%, while *P. fermentans* gradually dominated the fungi in CA group, accounting for 88.39%. LP treated silages showed the highest abundance of *W. anomalus* compared to other silages. In particular, the application of CAPF significantly reduce the abundance of yeast with the exception of *Trechispora* sp., which was most abundant in CAPF group and contributed to the differences compared to other silages (Figure 4D).

## Discussion

### Silage quality and aerobic stability

As the essential component of diet for animal fabrication, silage nutrition and hygienic quality are the major problem for farmers. Medicine food plant rich bioactive substances becomes in an interesting strategy to retard microbial contamination and deterioration *via* against a wide range of microorganisms, especially in the face of heavier restrictions on antibiotic use (Lv et al., 2011). In the present study, all additives with the exception of PF improved the silage quality of forage oat based on the lower NH_3_-N contents and greater residual of WSC in the term of 60 days of ensiling. It is worth noting that the application of citric acid inhibited the overall fermentation. Similar findings were reported by Lv et al. (2020) where high dose of citric acid (2%) inhibited the production of lactic acid and the growth of *Enterobacter*. Thus, from the perspective of anaerobic fermentation quality, *L. plantarum* a214, citric acid, alone or in combination with purple perilla were suitable additives.

Controlling the aerobic stability of forage silage is crucial for minimizing agricultural economic losses. With the opening of the silage, aerobic microorganisms began to grow and consume substances. Yeasts are generally the initiators of aerobic deterioration of silage, including fermentation acids and sugars assimilating species (Pahlow et al., 2003), resulting large of nutrition loss. Applying purple perilla in this study resulted in an increase in acetic acid and propionic acid content, a reduction in the yeast count and an improvement in aerobic stability. These effects were more evident in CAPF treated silages, thus highlighting a positive synergistic effect between citric acid and purple perilla in response to aerobic exposure. According to previous studies, purple perilla contains phenolic compounds, flavonoids, and terpenoids, such as perilla aldehyde, perilla alcohol, α-pinene, etc., which exhibit strongly inhibitory effects on a wide variety of fungi (Lim and Shin, 2014; Ahmed and Al-Zubaidy, 2020). In addition, the low-acidic environment enhanced the antimicrobial activity of phenols or terpenes (Reddy et al., 2020). It is interesting that even though the application of purple perilla combined with *L. plantarum* a214 or citric acid made a low-acidic environment, only CAPF group reduced the loss of nutrition of forage oat silage. This opposite results between LPPF and CAPF groups might be attributed to the component and concertation of fermentation acids (Muck et al., 2018). According to Pahlow et al. (2003), lactate-utilizing yeasts are the primary microorganisms responsible for initiating aerobic deterioration in most silages. The higher lactic acid content combined with high residues of WSC in LPPF group might counteracted the antimicrobial ability of purple perilla. However, the undissociated short-chain fatty acids more prevalent at low pH such as propionic and acetic acids are clearly more inhibitory to yeasts than the dissociated acids (Eklund, 1983). The higher content of these undissociated molecules in CAPF group could diffuse into the cell and lower the intracellular pH by releasing H+ ions, and rapidly kill the yeast cell. Similarly, citric acid, as undissociated acids, could also pass through the cell membrane easily and acidify the cytoplasm (Wemmenhove et al., 2016).

Higher concentrations of lactic acid and WSC residues in CK, LP, and LPPF groups, with a concomitant lower content of acetic acid, resulted in markedly increasing counts of yeast after 3 days of aerobic exposure. As a result, these silages had a higher rate of nutrition loss. Furthermore, yeast flora surviving the storage phase are highly related to the flora initiating aerobic deterioration (Pahlow et al., 2003), the higher counts of yeast in LP, LPPF, and CA groups during 60 days of ensiling might be one of the factors for lower aerobic stability.

The fermentation acids usually decreased as substrate by some aerobic microorganisms in response to aerobic exposure (Cantoia Júnior et al., 2020; Guan et al., 2020). Indeed, in the present study, the concentration of lactic and acetic acids sharply decreased in CK, LP, CA, and LPPF groups as the prolonged of aerobic exposure. It is noteworthy that there are no significant changes in PF and CAPF treatments within 3 days of aerobic exposure. The different response to aerobic exposure by the treatments may be due to the synthetic activities of acid assimilative microbes and facultative anaerobic or aerobic lactic acid bacteria (Jiang et al., 2020b). To confirm this hypothesis, we analyzed the bacterial and fungal communities in all silages, as well as their relative abundances at the genus and species levels.

### Microbial diversity of silage

In response to aerobic exposure, the OTUs, Chao1 and Shannon indices of bacteria increased in all silages. This indicated that the aerobic environment enhanced the growth of facultative anaerobes or aerobic bacteria, and increased the diversity of the bacterial community. This was similar to the observation of Guan et al. (2020) for Napier grass silage. In contrast to the results of bacterial diversity, the OTUs and Chao 1 indices of fungi decreased following aerobic exposure, which could be a consequence of some species dominating the fungi community in an aerobic environment. Noteworthily, no difference was observed in fungi diversity in purple perilla treated silages between 60-day ensiling and 3 days of aerobic exposure, indicating purple perilla could inhibit fungal growth. In addition, the beta diversity analysis revealed that *L. plantarum* a214 and citric acid were the factors that shaped the differences in bacteria and fungi in forage oat silages in the present study.

### Dynamic changes of bacterial communities

In general, successful fermentation of forage silages requires a fast growth of lactic acid bacteria to increase substantially in numbers and exert their effects. In the present study, *Lactobacillus* was the primary genus in all silages following 60-days ensiling, especially in *L. plantarum* a214, citric acid, or in combination with purple perilla groups. *Lactobacillus* species play a predominant role in lactic acid fermentation in silages. According to Lv et al. (2020) and Wang et al. (2019), *Lactobacillus* species and citric acid could decrease pH as starter at the early stage of silage, and the rapid acidification environment might increase the chance that the lactic acid bacteria outgrow undesirable species such as *Enterobacter* and *Clostridium*. However, the dominant species in treated silages underwent a change completely in this study. *L. plantarum* replaced other species as the primary population bacteria in *L. plantarum* a214 and LPPF treated silages. This finding was in agreement with the results of Yan et al. (2019), who reported *Lactobacillus* inoculum could increase substantially and dominated in silages. Differently, citric acid, alone or in combination with purple perilla, increased heterofermentative *Lactobacillus* species, which supported their higher acetic acid and lower lactic acid concentrations. It is worth noting that CAPF increased the relative abundance of *L. lindneri*. As an obligate heterofermentative species, *L. lindneri* could ferment sugars and produce large content of lactic acid, acetic acid, and diacetyl which had strongly anti-bacteria activity (Lanciotti et al., 2003; Liu et al., 2017). Especially, the substrate combined with citric acid could enhance its growth and metabolic capacity (Gobbetti and Corsetti, 1996). Therefore, the highest acetic acid concentration in CAPF silage during the ensiling might be due to the high relative abundance of *L. lindneri.*

Furthermore, the relative abundances of *Hafnia* and *Enterococcus* were also higher in CK and PF groups except *Lactobacillus* species compared with other treatments, respectively. *Hafnia* is a gram-negative genus of the family *Enterobacteriaceae*, which able to grow under anaerobic conditions as well as low pH environments (Wang et al., 2021). As undesirable bacteria, they compete with lactic acid bacteria for nutrients in both anaerobic and aerobic fermentation, resulting in large nutritional loss (Yang et al., 2021). In addition, some species of *Hafnia* such as *Hafnia alvei* could transform nitrogen from protein in silages into NH_3_ (Yang et al., 2021). Hence, the higher relative abundance of *Hafnia alvei* in CK groups, especially in aerobic exposure, could explain their higher DM loss and NH_3_-N.

After aerobic exposure, the compositions of the dominant bacteria in all silages were similar but with certain degrees of differences, except for the LPPF group, in which most of the *L. plantarum* were replaced by *Bacillus circulans*. According to Kung et al. (2018), *Bacilli* are one of the first groups of microorganisms to develop in silages after the aerobic spoilage process is initiated by yeasts. Even though they seem to be unable to initiate aerobic deterioration of silage, they can play a role in the later stages of aerobic deterioration after initial silage deterioration by yeast (Pahlow et al., 2003). The high abundance of *Bacillus* in LPPF group at 3 days of aerobic exposure might reveal its rapid aerobic deterioration.

### Dynamic changes of fungal communities

Yeasts are considered to be the most important group of microorganisms in aerobic deteriorated silage (Jiang et al., 2020b). In terms of 60 days ensiling, the yeast counts were similar in all silages, as most of them demonstrated low association to nutrition loss and fermentation characteristics (Supplementary Figure S1). Thus, we infer yeasts might not be the main factors affecting silage quality during the anaerobic phase.

In response to aerobic exposure, the relative abundance of *Pichia* species increased intensively in all silages except for the CAPF group, because of their highly lactate-assimilating abilities and strong affinities for sugars (Pahlow et al., 2003). *Pichia* is highly related to aerobic stability of silages. It is noteworthy that the dominated *Pichia* species of silages is different in silages, and *P. kudriavzevii* and *P. fermentans* were the predominant fungi in CK and CA silages, respectively. All of them were often detected in aerobic spoilage silages (Li and Nishino, 2011; Jiang et al., 2020a). According to Wang et al. (2018), *P. kudriavzevii* showed high tolerance to lactic and acetic acids. The high relative abundance of *P. kudriavzevii* made aerobic deterioration easier (Jiang et al., 2020a). Thus, inhibition of *P. kudriavzevii* might be an effective strategy for alleviating aerobic corruption in some instance. However, some non-Saccharomyces such as *P. fermentans* could utilize citric acid as an energy resource (Zhong et al., 2020). Especially, citric acid would accelerate the growth of *P. fermentans* in a sufficient oxygen environment (Zhong et al., 2021). Therefore, applying citric acid might be one of the factors that contribute to the higher abundance of *P. fermentans* and the less aerobically stable in CA group. A higher relative abundance of *W. anomalus* was detected in all *L. plantarum* a214 treated silages, which was in accordance with the report by Jiang et al. (2020a) that inoculation of *L. plantarum* could accelerate the growth of *W. anomalus*.

It is worth noting that there were no significantly changed of fungal composition in PF group between 60-day ensiling and 3 days of aerobic exposure, indicating purple perilla could alleviate the proliferation and metabolism of fungi during the aerobic exposure period. This could be a consequence of the antifungal ability of secondary metabolite in purple perilla, such as terpene, α-Farnesene, perilla ketone and so on (Lim and Shin, 2014; Ahmed and Al-Zubaidy, 2020). In particular, the application of purple perilla combined with citric acid decreased most of these fungi, except *Trechispora* sp. One possible reason is that there was a synergism of purple perilla and citric acid against these bacteria (Falleh et al., 2020; Reddy et al., 2020). The bioactive substance of purple perilla might disrupt cell membrane disruption, making fungal more vulnerable to the low pH environment generated by fermentation acids. On the other hand, the low pH environment in the CAPF group might enhance the hydrophobicity of bioactive substances, making it easier for them to dissolve in lipids in microbial cell membranes, and inhibiting the growth of microbial (Karatzas et al., 2001; Reddy et al., 2020).

## Conclusion

*Perilla frutescens* combined with citric acid (CAPF) could improve the silage quality of forage oat based on the lower dry matter loss, ammonia nitrogen and pH, and the higher residual of water-soluble carbohydrate in both ensiling and aerobic exposure. The application of CAPF clearly modulated the bacterial and fungal communities of forage oat silage and shifted the dominant bacteria species to heterofermentative *Lactobacillus* species, such as *L. lindneri* and *L. brevis*. In addition, *P. frutescens* has potential effects on forage oat silage spoilage by suppressing the growth of *Pichia* and *Wickerhamomyces* species, especially when combined with citric acid. Further attention should be paid to kinds of bioactive substances and the mechanisms of their effect on microbial and fungal communities in low-acidified environments.

## Data availability statement

The raw data supporting the conclusions of this article will be made available by the authors, without undue reservation. The data presented in the study are deposited in the NCBI, accession number PRJNA897720.

## Author contributions

XL, KN, and FY designed the study and wrote the manuscript. FC, YX, and LG performed the experiments. JX and YL conducted the statistical and bioinformatics analysis. KN and FY were involved in the revision of the manuscript. All authors contributed to the article and approved the submitted version.

## Funding

This research was supported by the earmarked fund for Modern Agro-Industry Technology Research System of China (grant number CARS-07-E-3) and the National Natural Science Foundation of China (grant numbers 3197140904).

## Conflict of interest

The authors declare that the research was conducted in the absence of any commercial or financial relationships that could be construed as a potential conflict of interest.

## Publisher’s note

All claims expressed in this article are solely those of the authors and do not necessarily represent those of their affiliated organizations, or those of the publisher, the editors and the reviewers. Any product that may be evaluated in this article, or claim that may be made by its manufacturer, is not guaranteed or endorsed by the publisher.

## Supplementary material

The Supplementary material for this article can be found online at: https://www.frontiersin.org/articles/10.3389/fmicb.2022.1053933/full#supplementary-material

## Footnotes

^1^ http://huttenhower.sph.harvard.edu/galaxy/

## References

[ref1] Ahmed H. M. Al-Zubaidy A. M. A. (2020). Exploring natural essential oil components and antibacterial activity of solvent extracts from twelve *Perilla frutescens* L. Genotypes. Arab. J. Chem. 13, 7390–7402. doi: 10.1016/j.arabjc.2020.08.016

[ref2] Cantoia Júnior R. Capucho E. Garcia T. M. Del Valle T. A. Campana M. Zilio E. M. C.. (2020). Lemongrass essential oil in sugarcane silage: fermentative profile, losses, chemical composition, and aerobic stability. Anim. Feed Sci. Technol. 260:114371. doi: 10.1016/j.anifeedsci.2019.114371

[ref3] Chan C.-L. Gan R.-Y. Shah N. P. Corke H. (2018). Polyphenols from selected dietary spices and medicinal herbs differentially affect common food-borne pathogenic bacteria and lactic acid bacteria. Food Control 92, 437–443. doi: 10.1016/j.foodcont.2018.05.032

[ref5] Drouin P. Tremblay J. Renaud J. Apper E. (2021). Microbiota succession during aerobic stability of maize silage inoculated with *Lentilactobacillus buchneri* NCIMB 40788 and *Lentilactobacillus hilgardii* CNCM-I-4785. Microbiology 10:e1153. doi: 10.1002/mbo3.1153, PMID: 33369186 PMC7885010

[ref6] Eklund T. (1983). The antimicrobial effect of dissociated and undissociated sorbic acid at different pH levels. J. Appl. Bacteriol. 54, 383–389. doi: 10.1111/j.1365-2672.1983.tb02632.x, PMID: 6409875

[ref7] Falleh H. Ben Jemaa M. Saada M. Ksouri R. (2020). Essential oils: a promising eco-friendly food preservative. Food Chem. 330:127268. doi: 10.1016/j.foodchem.2020.127268, PMID: 32540519

[ref8] Gobbetti M. Corsetti A. (1996). Co-metabolism of citrate and maltose by *lactobacillus brevis* subsp. lindneri CB1 citrate-negative strain: effect on growth, end-products and sourdough fermentation. Zeitschrift für Lebensmittel-Untersuchung und. Forschung 203, 82–87.

[ref9] Gomes A. L. M. Jacovaci F. A. Bolson D. C. Nussio L. G. Jobim C. C. Daniel J. L. P. (2019). Effects of light wilting and heterolactic inoculant on the formation of volatile organic compounds, fermentative losses and aerobic stability of oat silage. Anim. Feed Sci. Technol. 247, 194–198. doi: 10.1016/j.anifeedsci.2018.11.016

[ref10] Guan H. Shuai Y. Ran Q. Yan Y. Wang X. Li D.. (2020). The microbiome and metabolome of Napier grass silages prepared with screened lactic acid bacteria during ensiling and aerobic exposure. Anim. Feed Sci. Technol. 269:114673. doi: 10.1016/j.anifeedsci.2020.114673

[ref11] Hasan M. T. (2015). Official methods of analysis. 15th, Association of Official Analytical Chemists, Artington, Virginia, USA.

[ref12] Hussain A. Bose S. Wang J. H. Yadav M. K. Mahajan G. B. Kim H. (2016). Fermentation, a feasible strategy for enhancing bioactivity of herbal medicines. Food Res. Int. 81, 1–16. doi: 10.1016/j.foodres.2015.12.026

[ref13] Jia T. Yun Y. Yu Z. (2021). Propionic acid and sodium benzoate affected biogenic amine formation, microbial community, and quality of oat silage. Front. Microbiol. 12:750920. doi: 10.3389/fmicb.2021.750920, PMID: 34819922 PMC8606646

[ref14] Jiang D. Niu D. Zuo S. Tian P. Zheng M. Xu C. (2020a). Yeast population dynamics on air exposure in total mixed ration silage with sweet potato residue. Anim. Sci. J. 91:e13397. doi: 10.1111/asj.13397, PMID: 32484290

[ref15] Jiang D. Zheng M. L. Niu D. Z. Zuo S. S. Tian P. J. Li R. R.. (2020b). Effects of steam explosion pretreatment and *lactobacillus buchneri* inoculation on fungal community of unensiled and ensiled total mixed ration containing wheat straw during air exposure. J. Appl. Microbiol. 128, 675–687. doi: 10.1111/jam.14518, PMID: 31721404

[ref16] Kang M. Park J. Yoo S. (2019). Effect of clove powder on quality characteristics and shelf life of kimchi paste. Food Sci. Nutr. 7, 537–546. doi: 10.1002/fsn3.833, PMID: 30847132 PMC6392875

[ref17] Karatzas A. K. Kets E. Smid E. J. Bennik M. (2001). The combined action of carvacrol and high hydrostatic pressure on *listeria monocytogenes* Scott a. J. Appl. Microbiol. 90, 463–469. doi: 10.1046/j.1365-2672.2001.01266.x 11298243

[ref18] Kung L. Shaver R. D. Grant R. J. Schmidt R. J. (2018). Silage review: interpretation of chemical, microbial, and organoleptic components of silages. J. Dairy Sci. 101, 4020–4033. doi: 10.3168/jds.2017-13909, PMID: 29685275

[ref19] Lanciotti R. Patrignani F. Bagnolini F. Guerzoni M. E. Gardini F. (2003). Evaluation of diacetyl antimicrobial activity against *Escherichia coli, listeria monocytogenes* and *Staphylococcus aureus*. Food Microbiol. 20, 537–543. doi: 10.1016/S0740-0020(02)00159-4

[ref20] Li X. Chen F. Wang X. Sun L. Guo L. Xiong Y.. (2021a). Impacts of low temperature and ensiling period on the bacterial community of oat silage by SMRT. Microorganisms 9:274. doi: 10.3389/fmicb.2021.820011 33525587 PMC7910925

[ref21] Li X. Chen F. Xu J. Guo L. Xiong Y. Lin Y.. (2021b). Exploring the addition of herbal residues on fermentation quality, bacterial communities, and ruminal greenhouse gas emissions of paper mulberry silage. Front. Microbiol. 12:820011 35222315 10.3389/fmicb.2021.820011 PMC8874217

[ref22] Li D.-X. Ni K.-K. Zhang Y.-C. Lin Y.-L. Yang F.-Y. (2018). Influence of lactic acid bacteria, cellulase, cellulase-producing *Bacillus pumilus* and their combinations on alfalfa silage quality. J. Integr. Agric. 17, 2768–2782. doi: 10.1016/S2095-3119(18)62060-X

[ref23] Li Y. Nishino N. (2011). Effects of inoculation of *lactobacillus rhamnosus* and *lactobacillus buchneri* on fermentation, aerobic stability and microbial communities in whole crop corn silage. Grassl. Sci. 57, 184–191. doi: 10.1111/j.1744-697X.2011.00226.x

[ref24] Lim H. Shin S. (2014). Anti-bacillusand anti-shigellaactivities of the essential oil from *Perilla fruescensvar*. japonicaHara. J. Essent. Oil Bear. Plants 17, 309–316. doi: 10.1080/0972060X.2014.895157

[ref25] Liu J. Li L. Li B. Peters B. M. Deng Y. Xu Z.. (2017). First study on the formation and resuscitation of viable but nonculturable state and beer spoilage capability of *lactobacillus lindneri*. Microb. Pathog. 107, 219–224. doi: 10.1016/j.micpath.2017.03.043, PMID: 28377233

[ref26] Lv F. Liang H. Yuan Q. Li C. (2011). In vitro antimicrobial effects and mechanism of action of selected plant essential oil combinations against four food-related microorganisms. Food Res. Int. 44, 3057–3064. doi: 10.1016/j.foodres.2011.07.030

[ref27] Lv H. Pian R. Xing Y. Zhou W. Yang F. Chen X.. (2020). Effects of citric acid on fermentation characteristics and bacterial diversity of *Amomum villosum* silage. Bioresour. Technol. 307:123290. doi: 10.1016/j.biortech.2020.123290, PMID: 32265091

[ref28] Muck R. E. Nadeau E. M. G. Mcallister T. A. Contreras-Govea F. E. Santos M. C. Kung L. Jr. (2018). Silage review: recent advances and future uses of silage additives. J. Dairy Sci. 101, 3980–4000. doi: 10.3168/jds.2017-13839, PMID: 29685273

[ref29] Mugabe W. Shao T. Li J. Dong Z. Yuan X. (2020). Effect of hexanoic acid, *lactobacillus plantarum* and their combination on the aerobic stability of napier grass silage. J. Appl. Microbiol. 129, 823–831. doi: 10.1111/jam.14650, PMID: 32248604

[ref30] Pahlow G. Muck R. E. Driehuis F. Elferink S. J. Spoelstra S. F. (2003). “Microbiology of ensiling,” in Silage Science and Technology, eds. Buxton D. R. Muck R. E. Harrison J. H. 31–93. (Agronomy; No. 42).

[ref31] Reddy P. R. K. Elghandour M. M. M. Y. Salem A. Z. M. Yasaswini D. Reddy P. P. R. Reddy A. N.. (2020). Plant secondary metabolites as feed additives in calves for antimicrobial stewardship. Anim. Feed Sci. Technol. 264:114469. doi: 10.1016/j.anifeedsci.2020.114469

[ref32] Romero J. J. Joo Y. Park J. Tiezzi F. Gutierrez-Rodriguez E. Castillo M. S. (2018). Bacterial and fungal communities, fermentation, and aerobic stability of conventional hybrids and brown midrib hybrids ensiled at low moisture with or without a homo-and heterofermentative inoculant. J. Dairy Sci. 101, 3057–3076. doi: 10.3168/jds.2017-13754, PMID: 29395147

[ref33] Tao X. Wang S. Zhao J. Dong Z. Li J. Liu Q.. (2020). Effect of ensiling alfalfa with citric acid residue on fermentation quality and aerobic stability. Anim. Feed Sci. Technol. 269:114622. doi: 10.1016/j.anifeedsci.2020.114622

[ref34] Thomas T. A. (1977). An automated procedure for the determination of soluble carbohydrates in herbage. J. Sci. Food Agric. 28, 639–642. doi: 10.1002/jsfa.2740280711

[ref35] Wang H. Hao W. Ning T. Zheng M. Xu C. (2018). Characterization of culturable yeast species associating with whole crop corn and total mixed ration silage. Asian Australas J. Anim. Sci. 31, 198–207. doi: 10.5713/ajas.17.0183, PMID: 28728388 PMC5767501

[ref36] Wang Y. He L. Xing Y. Zheng Y. Zhou W. Pian R.. (2019). Dynamics of bacterial community and fermentation quality during ensiling of wilted and unwilted *moringa oleifera* leaf silage with or without lactic acid bacterial inoculants. mSphere 4, e00341–e00319. doi: 10.1128/mSphere.00341-19 31391277 PMC6686226

[ref37] Wang S. Li J. Zhao J. Dong Z. Dong D. Shao T. (2021). Silage fermentation characteristics and microbial diversity of alfalfa (*Medicago sativa* L.) in response to exogenous microbiota from temperate grasses. World J. Microbiol. Biotechnol. 37:204. doi: 10.1007/s11274-021-03155-7, PMID: 34677690

[ref38] Wemmenhove E. Van Valenberg H. J. Zwietering M. H. Van Hooijdonk T. C. Wells-Bennik M. H. (2016). Minimal inhibitory concentrations of undissociated lactic, acetic, citric and propionic acid for *listeria monocytogenes* under conditions relevant to cheese. Food Microbiol. 58, 63–67. doi: 10.1016/j.fm.2016.03.012, PMID: 27217360

[ref39] Yan Y. Li X. Guan H. Huang L. Ma X. Peng Y.. (2019). Microbial community and fermentation characteristic of Italian ryegrass silage prepared with corn Stover and lactic acid bacteria. Bioresour. Technol. 279, 166–173. doi: 10.1016/j.biortech.2019.01.107, PMID: 30721817

[ref40] Yang F. Wang Y. Zhao S. Feng C. Fan X. (2021). Dynamics of the fermentation products, residual non-structural carbohydrates, and bacterial communities of wilted and non-wilted alfalfa silage with and without *lactobacillus plantarum* inoculation. Front. Microbiol. 12:824229. doi: 10.3389/fmicb.2021.824229 35087507 PMC8788936

[ref41] Zacchino S. A. Butassi E. Liberto M. D. Raimondi M. Postigo A. Sortino M. (2017). Plant phenolics and terpenoids as adjuvants of antibacterial and antifungal drugs. Phytomedicine 37, 27–48. doi: 10.1016/j.phymed.2017.10.018, PMID: 29174958

[ref42] Zhao G. Q. Ju Z. L. Chai J. K. Jiao T. Jia Z. F. Casper D. P.. (2018). Effects of silage additives and varieties on fermentation quality, aerobic stability, and nutritive value of oat silage. J. Anim. Sci. 96, 3151–3160. doi: 10.1093/jas/sky207, PMID: 29846606 PMC6095438

[ref43] Zhong W. Chen T. Yang H. Li E. (2020). Isolation and selection of non-saccharomyces yeasts being capable of degrading citric acid and evaluation its effect on kiwifruit wine fermentation. Fermentation 6:25. doi: 10.3390/fermentation6010025

[ref44] Zhong W. Liu S. Yang H. Li E. (2021). Effect of selected yeast on physicochemical and oenological properties of blueberry wine fermented with citrate-degrading *Pichia fermentans*. LWT 145:111261. doi: 10.1016/j.lwt.2021.111261

