# Supplementary material for: Perilla frutescens as potential antimicrobial modifier to against forage oat silage spoilage
Source: Front Microbiol. 2022 Dec 20;13:1053933. doi: 10.3389/fmicb.2022.1053933 (PMC9807611; doi:10.3389/fmicb.2022.1053933)
Supplement: Supplementary file 1 [file Image_1.pdf]

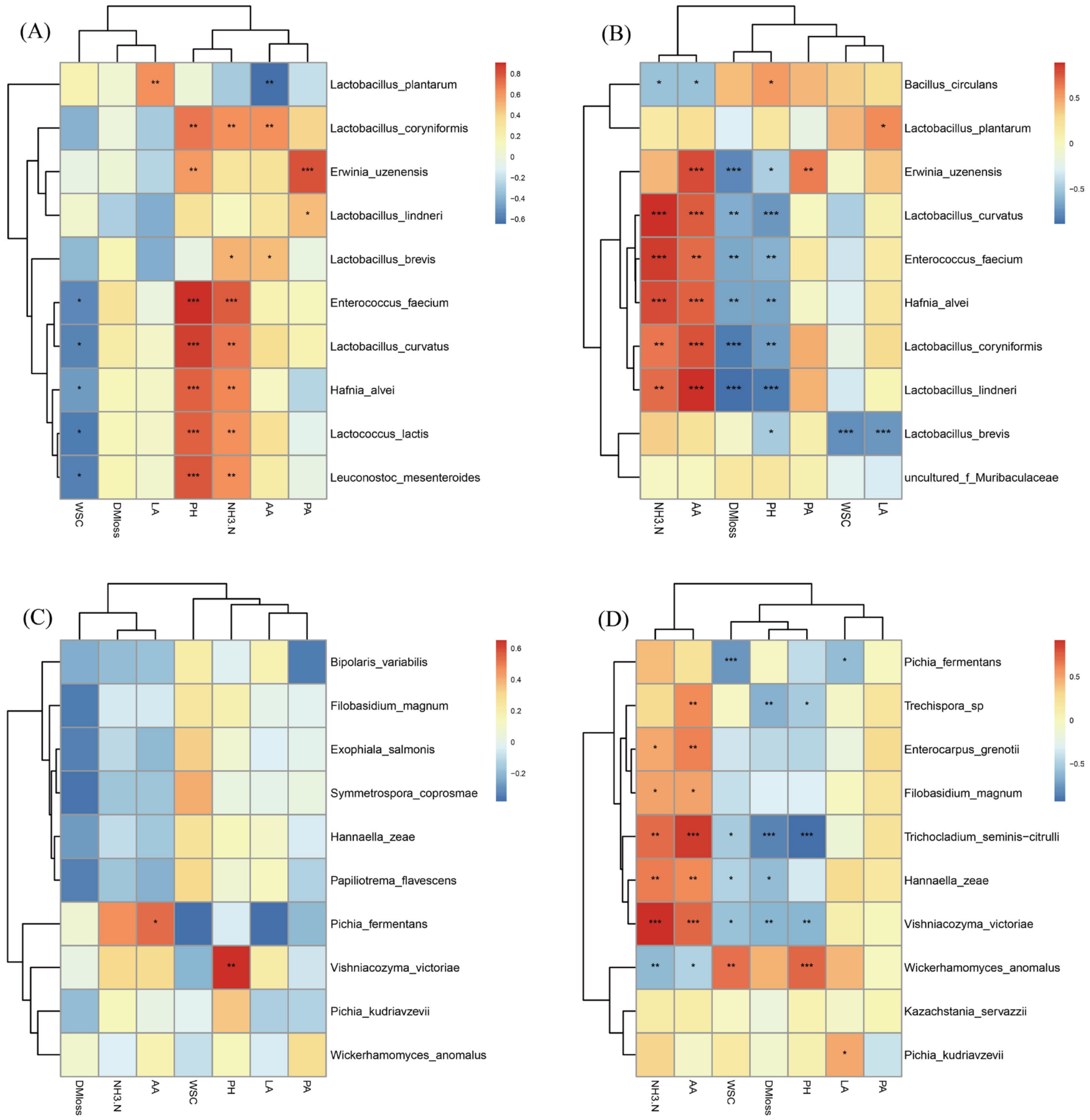

Figure 1S Correlation analyses between microbial community and fermentation products at species level. Bacteria species level on 60-day of ensiling (A) and 3 days of aerobic exposure (B); fungal species level on 60-day of ensiling (C) and 3 days of aerobic exposure (D). *, **, and *** represent $P < 0.05$, $P < 0.01$, and $P < 0.001$ respectively. CK, control; LP, *Lactobacillus plantarum* a214; CA, citric acid; PF, purple perilla; LPPF, purple perilla+*Lactobacillus plantarum* a214; CAPF, purple perilla+citric acid. LA, lactic acid; AA, acetic acid; PA, Propionic acid; NH_3-N, ammonia nitrogen; WSC, water-soluble carbohydrate; DM, dry matter.
